# Implementation of a SARS-CoV-2 genomic surveillance network as a strategy to face other health challenges in Peru

**DOI:** 10.17843/rpmesp.2026.431.15048

**Published:** 2026-03-02

**Authors:** Carlos Padilla-Rojas, Verónica Hurtado, Wendy Lizarraga, Víctor Jimenez-Vasquez, Iris S. Molina, Luis Barcena-Flores, Alicia Nuñez, Nieves Sevilla, Steve V. Acedo-Lazo, Estela Huamán-Angeles, Princesa Medrano, Kelly Izarra, Alexander Fajardo, Vanesa Dominguez, Karla Vasquez, Orson Mestanza, Francisco Ascue, Henri Bailon, Marco Galarza-Pérez, Omar Caceres-Rey, Roger V. Araujo-Castillo, Natalia Vargas-Herrera, Joseph Huayra, Gloria Arotinco-Garayar, Priscila Lope-Pari, Johanna Balbuena-Torres, Nancy Rojas-Serrano

**Affiliations:** 1 Innovation and Development Area, National Center for Public Health, National Institute of Health, Lima, Peru.; 2 Virology Unit, National Institute of Health, Lima, Peru.; 3 Strategic Interventions Unit, National Institute of Health, Lima, Peru.; 4 National Reference Laboratory for Vaccine-Preventable Viruses, National Center for Public Health, National Institute of Health, Lima, Peru.

**Keywords:** Public Health Surveillance, SARS-CoV-2, High-Throughput Nucleotide Sequencing, COVID-19

## Abstract

We present a complete description of the implementation of SARS-CoV-2 genomic surveillance during the COVID-19 pandemic in Peru, and the impact of applying this technology to other pathogens important for the country's public health. This aims to highlight the achievements, challenges, and lessons learned that allow for strengthening the response to current and future health challenges at the national level, as well as public health decisions. With the genomic surveillance initiative led by the National Institute of Health, 48,691 SARS-CoV-2 genomes from all regions of the country have been sequenced from 2020 to 2024, achieving the identification of eight variants of the virus and the designation of 25 new lineages within the Gamma, Delta, and Omicron variants. Genomic surveillance of SARS-CoV-2 has allowed for the timely identification of variants of epidemiological interest, monitoring the evolution of the virus in real-time, and using this information as a tool for public health decision-making. This experience subsequently provided the basis for monitoring other pathogens that have caused health emergencies in Peru and the world, such as dengue, influenza, and mpox.

## INTRODUCTION

Whole genome sequencing (WGS) of pathogens has currently consolidated as a fundamental tool in public health and constitutes an essential pillar in molecular epidemiology. Unlike traditional epidemiology, based on the description and follow-up of cases during an outbreak or epidemic, molecular epidemiology facilitates the characterization and early detection of emerging pathogen variants, as well as the analysis of their transmission patterns, expansion, and evolution over time ^1^. However, mass genomic sequencing is a highly specialized activity; therefore, prior to the pandemic, experience in these types of initiatives was primarily centered in developed countries ^2^.

In Peru, prior to the COVID-19 pandemic, the National Institute of Health (INS) had been using the Sanger method for the sequencing of pathogens of relevance to public health, such as the rabies virus ^3^ and varicella-zoster ^4^. In 2010, the platform was migrated toward next-generation sequencing (NGS) to meet the need initially sparked by the influenza pandemic ^5^. In March 2020, faced with the need to improve the detection of SARS-CoV-2 positive cases, researchers from the National Institute of Health (INS) successfully sequenced the first case of SARS-CoV-2 in Peru through metagenomic sequencing ^6^. Although an in-house method based on RT-qPCR was developed, the genomic surveillance capacity was limited and depended on funded projects and the INS budget. In this context, the INS formed a genomic surveillance team to study virus mutations and carry out the initiative in a coordinated and effective manner.

From the confirmation of the first case in Peru until the end of 2024, the INS promoted national genomic surveillance, successfully sequencing more than 48,000 SARS-CoV-2 genomes to date. This article highlights the experience, challenges, and achievements in the genomic surveillance of SARS-CoV-2 from 2020 to 2024 and how its implementation to respond to the COVID-19 pandemic was important for other public health challenges that arrived later. This aims to highlight lessons learned that allow for strengthening the response to current and future health challenges at the national level, as well as public health decisions.

## DESCRIPTION OF THE IMPLEMENTATION OF THE GENOMIC SURVEILLANCE NETWORK

The INS, in coordination with the Ministry of Health (MINSA), designed the SARS-CoV-2 genomic surveillance plan during the health emergency (Registry No. 4814-2021) ^7^, which included coordination with 25 Regional Reference Laboratories (LRR), private laboratories, Directorates of Integrated Health Networks (DIRIS), and Regional Health Directorates or Managements (DIRESAS/GERESAS); covering everything from the collection and transport of clinical samples to the decentralization of the test.

The implementation of genomic surveillance followed these stages: (i) development and approval of the protocol, (ii) coordination with the LRRs, (iii) transport of samples to the INS, (iv) genomic sequencing and bioinformatic analysis, (v) publication of genomes in public databases, and (vi) reporting of results to health authorities for public health decision-making.

**Figure 1:**
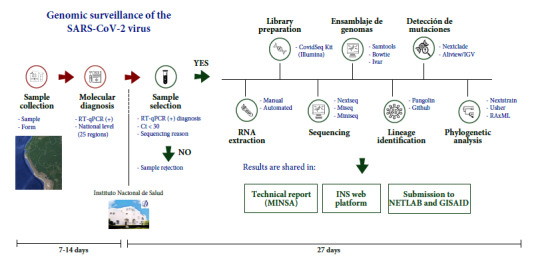
Workflow of the genomic surveillance of SARS-CoV-2 carried out at the INS of Peru. Sample collection is performed by the reference laboratories of the 25 regions of the country, DIRIS, DIRESAS, and laboratories of private institutions.

The genomic surveillance process requires a period of 15 days for the issuance of results, from case detection to characterization. This timeframe is due to the complexity of the procedure, difficulties in sample transport due to Peru's topography, and manual editing of the genomes, a crucial stage to ensure data quality. The complete workflow is described in figure 1.

INS: National Institute of Health, MINSA: Ministry of Health, NETLAB: Information System of the National Network of Public Health Laboratories in Peru, GISAID: Global Initiative on Sharing all Influenza Data.

Samples sent to the INS were selected at the LRR level, where staff chose those with a positive RT-qPCR diagnosis and a Ct value < 30. Although most samples were selected randomly, prioritization was also considered if the sample came from an outbreak, an overseas traveler (with a positive diagnosis in the last 14 days), or a person with reinfections, patients with unusual symptoms, or those hospitalized in the intensive care unit, reaching 10 samples per region. Samples with poor labeling, spills, insufficient volume, or non-compliance with transport and preservation standards according to current regulations were excluded.

For the extraction of genetic material, automated platforms were used: Quick-DNA/RNA Viral MagBead Kit on the OT-2 automated equipment (Opentrons) for 48 samples, and Prepito Viral NA Body Fluid Kit on the Chemagic® 360 and Prepito-D equipment for 96 and 12 samples, respectively. Subsequently, library preparation was performed using the COVIDSeq Test kit (Illumina Inc.), followed by sequencing on NextSeq 550, MiSeq, and MiniSeq equipment using paired-end reads of 150 nt (300 cycles). Sequencing primers were obtained from the Artic Network GitHub (https://github.com/artic-network/artic-ncov2019/tree/master/primer_schemes/nCoV-2019).

Files obtained from sequencing were analyzed to obtain the full genome of the virus. First, quality control was performed using the FastQC program (https://www.bioinformatics.babraham.ac.uk/projects/fastqc/). Reads were mapped to the SARS-CoV-2 Wuhan-Hu-1 reference genome (NC_045512) using Samtools, BWA, and iVar, while the identification of variants and lineages was performed with the Phylogenetic Assignment of Named Global Outbreak Lineages (Pangolin) (https://github.com/cov-lineages/pangolin), and nucleotide and amino acid mutations were detected with Nextclade (https://clades.nextstrain.org/). Genomes were manually reviewed with Aliview and IGV to be subsequently analyzed through the construction of phylogenies with Nextstrain (https://nextstrain.org/). The resulting genomes were deposited in the public database Global Initiative on Sharing All Influenza Data (GISAID) (https://gisaid.org/) for use by the scientific community. Furthermore, genomic surveillance results were registered in the Netlab.v2.0 system (https://netlabv2.ins.gob.pe/) and published on the INS website (https://www.gob.pe/40668-conocer-la-secuenciacion-genomica-del-covid-19-en-el-peru) through an interactive platform providing data on circulating lineages and variants, as well as their geographic distribution. All these data were compiled into weekly technical reports issued to MINSA, which are available on the unique digital platform of the Peruvian state (https://repositorio.ins.gob.pe/).

Regarding the administrative considerations of this initiative, it should be noted that samples were collected within the framework of the "COVID-19 Clinical-Epidemiological Investigation Form," present in the "Technical Guide for Investigation and Control of COVID-19 Outbreaks in Institutions with Captive Populations" approved by Ministerial Resolution No. 440-2022/MINSA.

## SCALING UP GENOMIC SURVEILLANCE

As mentioned, during 2020, the capacity to perform genomic sequencing in our country was very limited and depended mainly on the internal budget of the INS. In this sense, the possibility of conducting exhaustive surveillance was very restricted. In March 2021, a national plan was developed, which contemplated increasing the capacity to sequence 384 SARS-CoV-2 samples weekly from all regions of the country. In 2022, this capacity increased to 912 samples per week due to the hiring of more professionals and the acquisition of equipment. This was considered necessary because regions requested local information, as by that time the genetic dispersion of the virus was broad and each region had different transmission dynamics.

It should be highlighted that in the most remote regions, which required genomic information but whose care was complicated by sample transport difficulties, the Gen Maskaq mobile laboratory was implemented—a mobile laboratory adapted in a motorized vehicle—whose objective was to perform in situ genomic surveillance in regions with a significant increase in COVID-19 cases. This prefabricated module is equipped with an electrical network, ventilation, sanitary facilities, and furniture, in addition to having the necessary laboratory equipment to perform the genomic sequencing process and separate areas to maintain unidirectional flow (supplementary material 1).

Additionally, at the end of 2021, the INS decentralized SARS-CoV-2 genomic surveillance to three key regions: Piura, Cusco, and Junín, in collaboration with their LRRs. This allowed for the training of a team of professionals in the NGS technique in each of these regions, enabling the processing of up to 48 samples weekly in each LRR. The decentralization was a success, thanks to the existing assay methods, protocols, and health directives that facilitated effective replication.

**Figure 2:**
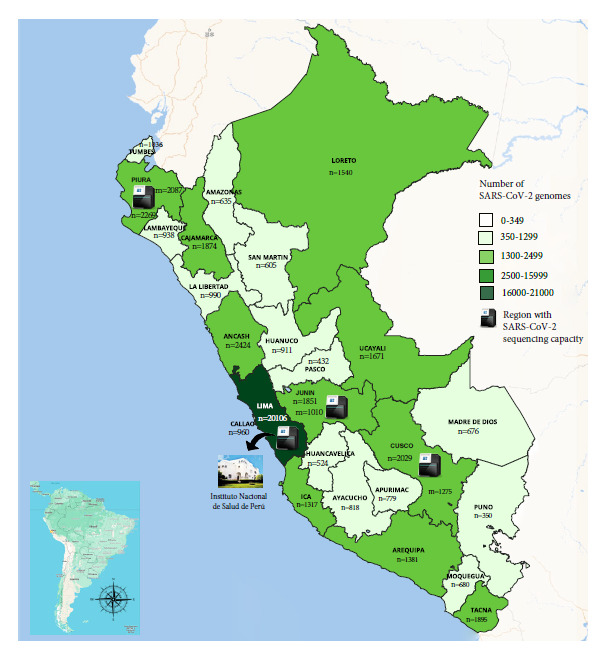
SARS-CoV-2 genomes obtained as a product of the genomic surveillance carried out by the National Institute of Health (INS) from 2020 to 2024. The number of genomes sequenced by region (n) at the INS is detailed. Likewise, a sequencer icon highlights the regions where genomic surveillance is performed, detailing the number of genomes processed by each region (m).

**Figure 3:**
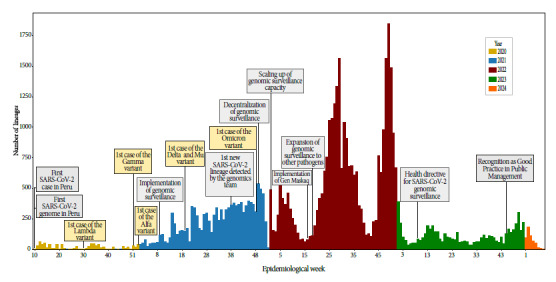
SARS-CoV-2 lineages identified by epidemiological week (EW) from 2020 to 2024 at the National Institute of Health of Peru. The graph illustrates the number of lineages identified from EW 10 of 2020 to EW 08 of 2024. It highlights the most important events in the implementation of genomic surveillance during the COVID-19 pandemic. Each year is represented by a different color: 2020 (yellow), 2021 (blue), 2022 (red), 2023 (green), and 2024 (orange).

In total, the INS managed to characterize 48,691 genomes from all 25 regions of the country until April 9, 2024 (figure 2), with Lima being the region with the highest number of sequenced cases. Progressively, the INS increased its genomic sequencing capacity, from producing 851 genomes in 2020 to 12,246 genomes the following year thanks to the surveillance plan; this figure increased exponentially to 29,157 genomes in 2022, partly due to the decentralization and scaling up of processes already described. In 2023, despite the decrease in cases, 5,894 genomes were processed, and in 2024, 543 genomes have been sequenced up to April of that year (figure 3). Between 2021 and 2022, the LRRs sequenced a total of 4,372 genomes.

## MEMBERSHIP IN INTERNATIONAL NETWORKS

The technical reports for SARS-CoV-2 genomic surveillance are available at: https://www.gob.pe/institucion/ins/colecciones/15034-informes-tecnicos-de-variantes-y-linajes-del-virus-sars-cov-2. At the time, they constituted important inputs for MINSA decision-making. Just as a genomic analysis network was formed between the INS and some regions within the country, Peru participated in international genomic analysis networks of the continent, particularly the Regional Network for Genomic Surveillance of COVID-19 in the Americas (COVIGEN) and the Regional Network for Genomic Surveillance of Respiratory Viruses (RESVIGEN), among others.

In this sense, scientific dissemination has been fundamental for creating collaboration networks and promoting research (supplementary material 2), in events organized by the INS of Colombia, the Federal Network of Genomics and Bioinformatics in Argentina, the Andean Health Organization and the Hipolito Unanue Convention (ORAS-CONHU), Seoul Clinical Laboratories of South Korea, and the Pan American Health Organization.

## IMPACT OF MASSIVE SARS-CoV-2 SEQUENCING IN PERU

To date, 55,280 SARS-CoV-2 genomes have been generated in Peru, 88% of which are the result of the INS's work, making it, according to GISAID, the second country in South America with the highest reported SARS-CoV-2 genome sequencing, surpassed only by Brazil ^8^.

**Figure 4:**
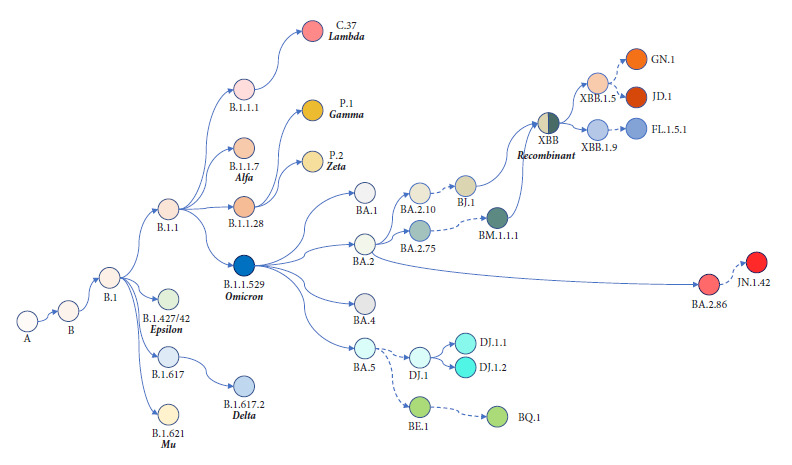
Graphical representation of the main variants and lineages of SARS-CoV-2 identified in Peru from 2020 to 2024. The evolution of the SARS-CoV-2 virus and the emergence of variants of concern, interest, and under surveillance in Peru are illustrated. Direct descent of a lineage is represented by solid lines, while dotted lines denote that multiple intermediate lineages exist in its evolution. Lineages or variants with higher prevalence or those causing epidemiological waves are represented with a blue border.

Throughout the implementation process of SARS-CoV-2 genomic surveillance in Peru, the INS detected the circulation of 8 variants of SARS-CoV-2 in the country from 2020 to 2024: 4 variants of concern (VOC) Alpha (B.1.1.7), Gamma (P.1), Delta (B.1.617), and Omicron (B.1.1.529); 2 variants of interest (VOI) Lambda (C.37) and Mu (B.1.621); and 2 variants under monitoring (VUM) Epsilon (B.1.429) and Zeta (P.2.). It should be noted that at the end of 2022, recombinant lineage cases within the Omicron variant were detected for the first time. Some lineages such as DJ.1, GN.1, JD.1, BA.2.86, and JN.1.42 have caused concern in Peru due to their high prevalence and rapid dispersion; however, they have not been designated as variants by the World Health Organization (WHO). Further details on the detected SARS-CoV-2 variants and lineages can be found in figure 4.

At the beginning of the pandemic, a wide circulation of the B.1.1 lineage was detected, which was the most persistent, especially in Lima and Callao ^9^. This lineage would give rise to the first variant of concern designated by the WHO, named Alpha (B.1.1.7), which was more predominant in Europe; only a few cases were reported in Peru ^10^, related to limited genomic characterization and the arrival of other variants of interest such as Lambda (C.37) and concern such as Gamma (P.1) that dispersed during the second epidemiological wave in Peru ^11^^,^^12^. During this period in 2021, several sublineages of the Gamma variant were detected for the first time, named P.1.12, P.1.12.1, and P.1.7.1, circulating in countries such as the United States, Brazil, and Chile.

In the second quarter of 2021, VOC Delta (B.1.617.2) and VOI Mu (B.1.621) emerged. During this period, six sublineages were detected within the wide diversity of Delta; these were designated as AY.26.1, AY.3.2, AY.102.2, AY.102.1, AY.119.1, and AY.25.1.1, some of them apparently having been generated in Peru, while others dispersed to Chile, the United States, Brazil, Colombia, Spain, and Costa Rica. At the end of 2021, Delta was displaced by VOC Omicron (B.1.1.529), causing the third epidemiological wave in Peru in early 2022.

In 2022, a wide diversity and sequential displacement of Omicron was observed, highlighting its lineages BA.1, BA.2, BA.4, and BA.5, which were detected in Peru successively. This displacement between lineages, observed globally, is due to competition between variants with advantages in transmissibility and/or greater immune escape ^13^. From BA.5, we saw the emergence of its descendants such as DJ.1, DJ.2, and BQ.1 ^14^. Globally, BA.5 has demonstrated significant convergent evolution, with numerous shared mutations in the spike gene ^15^, besides being highly transmissible and capable of evading previous immunity ^16^. Within Omicron, we detected 15 descendant lineages of BA.1, BA.2, DJ.1, BQ.1, and GN.1. At the end of 2022, the presence of the recombinant Omicron lineage XBB was detected, a product of the combination of lineages BA.2.10.1 and BA.2.75. Although the XBB recombinant shows a greater capacity for immune evasion compared to previous Omicron lineages, there is no evidence that it causes more severe disease (https://boletin.ins.gob.pe/informacion-institucional-n6/). In 2023, the XBB recombinant predominated until epidemiological week SE 06, when it began to decline slowly, being displaced by XBB.1.5 lineages and their descendants. At the end of 2023, the JN.1 lineage, a direct descendant of BA.2.86.1, began to increase rapidly, remaining until SE 08 of 2024, from which point only the sublineages JN.1 and JN.1.42 prevailed as the most frequent.

In total, the INS Genomic Surveillance team proposed 62 new SARS-CoV-2 lineages from 2021 to 2024, achieving approval and designation for 25 by the Pangolin international committee (https://github.com/cov-lineages/pango-designation). These belong to Gamma (P.1.12, P.1.12.1, P.1.7.1), Delta (AY.26.1, AY.3.2, AY.102.2, AY.102.1, AY.119.1, AY.25.1.1), Omicron (BA.1.22, BC.2, BA.1.15.3, BA.2.53, BG.1, BA.2.60, BG.3, BG.6, BG.7, DJ.1.2, DJ.1.1.1, DJ.1.3, BQ.1.1.50, BQ.1.11.1, GN.1.4), and Recombinant (XAM). Their characteristics are described in detail in supplementary material 3. This reflects the INS's contribution to the global genomic knowledge of the virus.

## GENOMIC SURVEILLANCE OF OTHER PATHOGENS OF PUBLIC HEALTH INTEREST

After the SARS-CoV-2 pandemic, Peru has faced outbreaks of various infectious diseases, including dengue, influenza, and mpox. Having implemented national capacities for the genomic surveillance of pathogens, the skills acquired could be applied to other epidemics.

### Mpox

In 2022, the WHO declared an outbreak of the mpox virus, which infected 3,800 people in Peru and caused 20 deaths in 21 regions of the country, mostly in people with a weakened immune system ^17^. As a response to the outbreak, the INS, through the genomic surveillance team, characterized the genome of the first confirmed case of mpox in Peru in June 2022; subsequently, sequencing of confirmed cases at the national level continued, with the highest percentage of characterized genomes coming from the regions of Lima, Callao, La Libertad, and Arequipa. During the outbreak and subsequent years of surveillance, the INS was the only Peruvian institution that performed genomic characterization of mpox, reporting 575 genomes belonging to clade IIb, with the predominant lineage being B.1.6, native to Peru, which dispersed widely throughout the country (https://github.com/mpxv-lineages/lineage-designation/issues/9). The fact of having produced a country-specific lineage that became predominant indicates that the epidemic in Peru possibly had a greater magnitude than reported.

### Influenza

In 2023, systematic sequencing of the influenza virus began as part of the implementation of a genomic monitoring system, given the virus's high capacity for mutation and genetic reassortment. Initial results allowed for the identification of circulating type A influenza viruses, specifically subtypes A(H1N1)pdm09 and A(H3N2), as well as the B/Victoria lineage belonging to type B influenza. On the other hand, phylogenetic analyses revealed evolutionary patterns of the virus over time, evidencing the formation of specific genetic clades according to the circulation period. Currently, quarterly genomic surveillance of the human influenza virus is performed.

In the context of the health emergency for highly pathogenic avian influenza (H5N1) registered in Peru during 2023, genetic characterization was achieved for 13 isolates of the H5N1 subtype obtained from wild birds and mammals, mainly in coastal ecosystems. Phylogenetic analyses established that all viral sequences belonged to clade 2.3.4.4b. This work was developed in coordination with the National Forestry and Wildlife Service and the National Agricultural Health Service, entities responsible for the collection and transport of biological samples for molecular processing ^18^.

### Dengue

The INS has been participating in the response to the 2023 dengue virus outbreaks, managing to characterize 289 samples. However, it was in 2024 that the INS implemented quarterly genomic surveillance of the dengue virus. Within the surveillance framework, 1,184 samples were characterized via NGS in 2024. This implementation has allowed for real-time monitoring of genotypes and mutations present in this virus.

Initial surveillance results have identified circulating genotypes within the 3 dengue serotypes: genotype V (DENV-1), Cosmopolitan genotype (DENV-2), and genotype III (DENV-3). These results coincide with those previously reported from 2018 to 2022 ^19^. During 2023, the Cosmopolitan genotype of the DENV-2 serotype was identified, showing a wide geographic distribution across different regions of the country with a higher proportion of cases. However, a change was evidenced by the increase of genotype III of the DENV-3 serotype and particularly the growth of a new clade, a finding reported in the second quarter of 2024.

Unlike the identification of serotypes performed through serological tests, to date, the characterization of dengue genotypes and lineages can only be performed by genomic sequencing and subsequent phylogenetic analysis to evaluate the formation and distribution of clades. Continuing with national genomic surveillance of the dengue virus will allow for a better understanding of its epidemiological behavior in the country.

## RECOMMENDATIONS

Based on the experience acquired with SARS-CoV-2 genomic surveillance, the proper functioning of the Regional Reference Laboratories and the INS performing genomic surveillance must be maintained through adequate funding and trained personnel to face future health emergencies. We consider it vital to maintain the use of next-generation sequencing to identify variants and lineages of viral strains that pose a risk to national public health.

In this sense, we must promote the decentralization of sequencing to more regions of the country so they can conduct timely surveillance of circulating pathogens in their region and address regional public health problems. It is important to highlight that all genomic information collected as a result of this surveillance must be available and open-access to the scientific community for the benefit of the entire population.

## CONCLUSIONS

Since the implementation of SARS-CoV-2 genomic surveillance in 2020, it has been possible to monitor the entry of new variants and the emergence of viral lineages, identifying the circulation of 465 lineages in our territory and registering 25 native to our country. This genomic surveillance proved its utility during the COVID-19 pandemic, allowing for an understanding of the virus's dispersion and evolution in our country.

The implementation of surveillance for SARS-CoV-2 was the response to the need for more precise information on pathogens, which is why it was considered necessary to expand it to other diseases causing outbreaks. Thus, it was initially implemented for mpox, resulting in the assignment of 575 samples within clade IIb and identifying that lineage B.1.6, native to Peru, was the most predominant in the country. Subsequently, surveillance was used during the risk identification of the introduction of Avian Influenza, finding that clade 2.3.4.4b of the highly pathogenic group was circulating in birds and mammals along the Peruvian coast. Finally, in the dengue epidemic, genotypes V (DENV-1), Cosmopolitan (DENV-2), and III (DENV-3) were identified, and serotype succession was analyzed, observing that the DENV-2 serotype, most frequent in 2023, was displaced by the DENV-3 serotype in 2024.

The main challenge in health emergencies due to epidemics, at the time of implementing genomic surveillance, is ensuring the necessary funds to implement a sample transport system, sequencing supplies, trained personnel, and infrastructure improvement. The experience acquired in SARS-CoV-2 genomic surveillance in Peru could serve as a model for subsequent implementations during outbreaks or epidemics such as those mentioned. It is crucial to standardize and unify health information in the country and connect it with the genomic surveillance system to improve response capacity. The threat of pandemics has fostered fluid collaboration between sectors, which must be promoted and prioritized to face new public health threats.

We consider that the implementation of genomic surveillance for pathogens of public health interest has been a success in our country. As a result of all the outcomes obtained in the implementation, multiple recognitions have been received. In 2023, the genomic surveillance practice was recognized as a Good Practice in Public Management by the organization Ciudadanos al Día and obtained third place in the XVIII National Meeting of Experiences in Quality Improvement in Health organized by MINSA.

Despite the success of genomic surveillance, multiple challenges persist that must be addressed to improve the response capacity for the next health emergency. Since the beginning of the pandemic, it has been evident that the country's topography complicates sample transport from various districts and provinces, causing delays in sending samples to the INS. This delay has been exacerbated by logistical and budgetary issues, making the need for a more efficient system for sample transport even more urgent. Likewise, it is necessary to sensitize the population about the importance of genomic surveillance, as it allows for the identification of new variants and monitoring of their evolution, helping to implement effective control strategies and strengthen the response to future health crises. Therefore, it is extremely important to ensure the necessary budget to continue this crucial activity, in order to continue implementing it for other upcoming public health threats.
