# Supplementary material for: Implementation of a SARS-CoV-2 genomic surveillance network as a strategy to face other health challenges in Peru
Source: Rev Peru Med Exp Salud Publica. 2026 Mar 2;43(1):110–8. doi: 10.17843/rpmesp.2026.431.15048 (PMC13245991; doi:10.17843/rpmesp.2026.431.15048)
Supplement: Supplementary material. — Available in the electronic version of the RPMESP. [file rpmesp-43-01-15048-s001.docx]

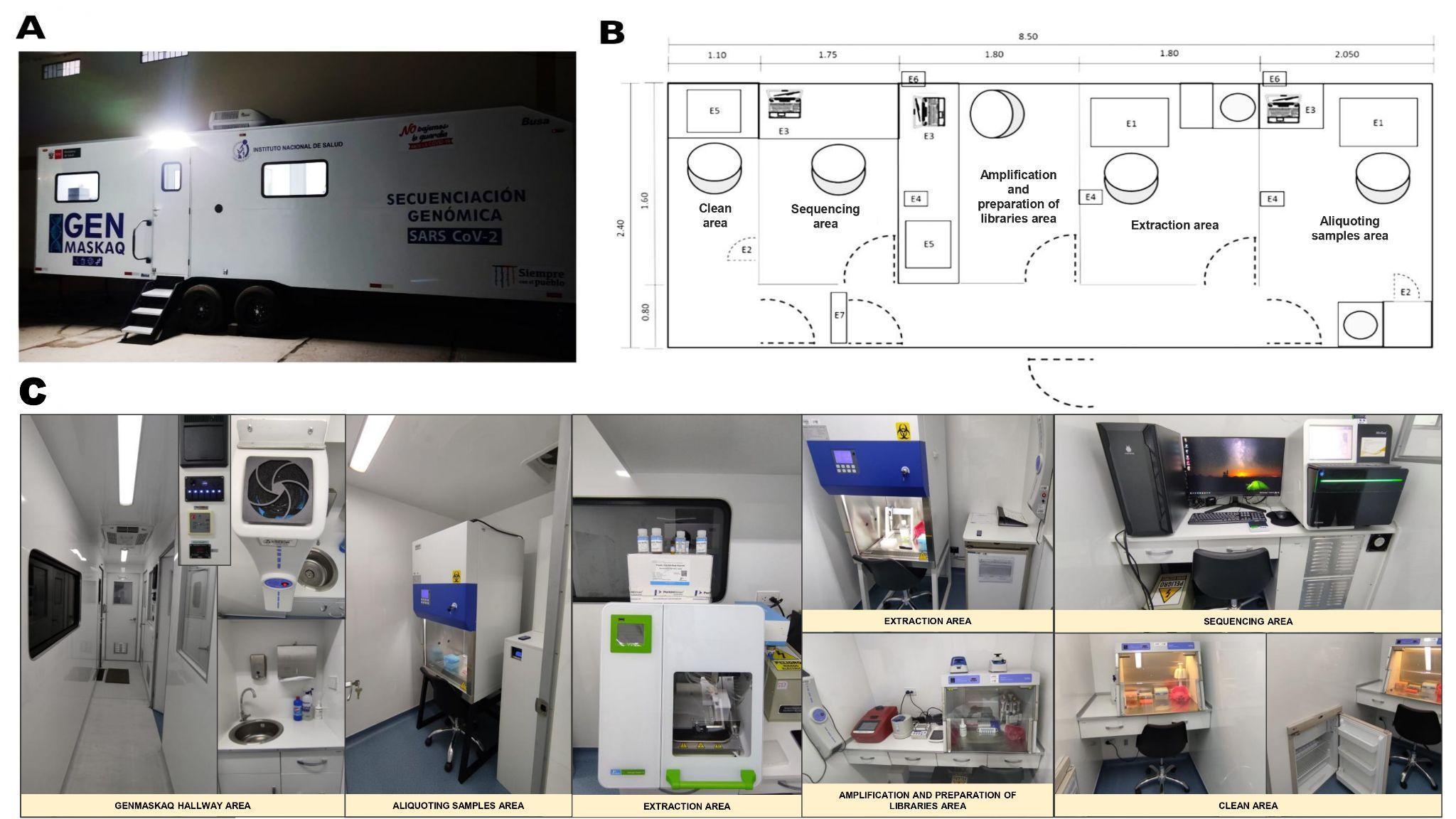


**Material suplementario 1.-** A) Laboratorio móvil para secuenciación genómica de SARS-CoV-2 “Gen Maskaq”, diseñado e implementado por el Instituto Nacional de Salud. B) Diseño interior del laboratorio móvil “Gen Maskaq” elaborado por el equipo técnico del Centro Nacional de Salud Pública del Instituto Nacional de Salud. C) Fotografía de las áreas internas del laboratorio móvil “Gen Maskaq”.

**Material suplementario 2.-** Tabla resumen de las publicaciones obtenidas por el equipo de Vigilancia Genómica de SARS-CoV-2 del Instituto Nacional de Salud del Perú desde el año 2021 al 2024.

| **Año** | **Publicación** | **Revista científica** |
| --- | --- | --- |
| 2020 | Genomic analysis reveals local transmission of SARS-CoV-2 in early pandemic phase in Peru. | *bioRxiv* |
| 2020 | Near-complete genome sequence of a 2019 novel coronavirus (SARS-CoV-2) strain causing a COVID-19 case in Peru. | *Microbiology Resource Announcements* |
| 2021 | Genomic analysis reveals a rapid spread and predominance of lambda (C. 37) SARS‐COV‐2 lineage in Peru despite circulation of variants of concern. | *Journal of Medical Virology* |
| 2021 | Near-Complete Genome Sequence of a SARS-CoV-2 VOC 202012/01 Strain in Peru. | *Microbiology Resource Announcements* |
| 2022 | SARS-CoV-2 Lambda and Gamma variants competition in Peru, a country with high seroprevalence. | *The Lancet Regional Health-Americas* |
| 2022 | Reinfecciones por SARS-CoV-2 durante la segunda ola pandémica en Iquitos, Perú. | *Revista del Cuerpo Médico Hospital Nacional Almanzor Aguinaga Asenjo* |
| 2022 | Genomic surveillance of the Lambda SARS‐CoV‐2 variant in a global phylogenetic context. | *Journal of Medical Virology* |
| 2022 | Confirmed severe acute respiratory syndrome coronavirus 2 reinfections after a second wave with predominance of lambda in Lima and Callao, Peru. | *In Open Forum Infectious Diseases* |
| 2024 | Dispersion of SARS-CoV-2 lineage BA.5.1.25 and its descendants in Peru during two COVID-19 waves in 2022. | *Genomics & Informatics* |

**Material suplementario 3.-** Linajes de SARS-CoV-2 propuestos y asignados en PANGOLIN, por el equipo de Vigilancia Genómica del Instituto Nacional de Salud del Perú desde el año 2021 al 2024.

| **Año** | **Variante** | **Linaje** | **Mutaciones asociadas** | **Países circulantes*** | **Link Pangolin** |
| --- | --- | --- | --- | --- | --- |
| 2021 | Gamma | P.1.12 | S: R246G, N679K | Perú, Estados Unidos, Brasil, Chile, otros. | <https://github.com/cov-lineages/pango-designation/issues/212> |
| 2021 | Delta | AY.26.1 | S: E484Q, T1009S  29769C>T | Perú, otros. | <https://github.com/cov-lineages/pango-designation/issues/278> |
| 2021 | Gamma | P.1.12.1 | S: N679K, R246G, T284I | Perú, otros. | <https://github.com/cov-lineages/pango-designation/issues/282> |
| 2021 | Delta | AY.3.2 | S: T259I | Perú, otros. | <https://github.com/cov-lineages/pango-designation/issues/312> |
| 2021 | Delta | AY.102.2 | E: L51I | Perú, otros. | <https://github.com/cov-lineages/pango-designation/issues/330> |
| 2021 | Delta | AY.102.1 | S: G1219V | Perú, otros. | <https://github.com/cov-lineages/pango-designation/issues/325> |
| 2021 | Delta | AY.119.1 | Orf1ab: A5692S  S: I834T  Orf3a: A54S  N: K373N | Perú, Chile, Estados Unidos, Brasil, Colombia, España, Costa Rica, otros. | <https://github.com/cov-lineages/pango-designation/issues/336> |
| 2021 | Delta | AY.25.1.1 | S: D796Y | Perú, Estados Unidos, otros. | <https://github.com/cov-lineages/pango-designation/issues/355> |
| 2021 | Gamma | P.1.7.1 | Orf1ab: T3284I Orf1ab: L3677F  3049T>C | Perú, Estados Unidos, otros. | <https://github.com/cov-lineages/pango-designation/issues/326> |
| 2022 | Ómicron | BA.1.22 | Orf1ab: E973K | Perú, otros. | <https://github.com/cov-lineages/pango-designation/issues/529> |
| 2022 | Ómicron | BC.2 | Orf1b: V1961I | Perú, Canadá, otros. | <https://github.com/cov-lineages/pango-designation/issues/570> |
| 2022 | Ómicron | BA.1.15.3 | Orf1a: H1113Y | Perú, Estados Unidos, Reino Unido, otros. | <https://github.com/cov-lineages/pango-designation/issues/573> |
| 2022 | Ómicron | BA.2.53 | S: Y449N  N: T135I  8605C>T, 25306T>C | Dinamarca, Perú, Reino Unido, España, Francia, Suecia, Alemania, Irlanda, otros. | <https://github.com/cov-lineages/pango-designation/issues/651> |
| 2022 | Ómicron | BG.1 | Orf1a: N2065D | Perú, Estados Unidos, Reino Unido, otros. | <https://github.com/cov-lineages/pango-designation/issues/679> |
| 2022 | Ómicron | BA.2.60 | S: V1129I  Orf1a: T551S | Perú, Estados Unidos, Chile, Alemania, otros. | <https://github.com/cov-lineages/pango-designation/issues/683> |
| 2022 | Ómicron | BG.3 | Orf1a: T2967A | Perú, Estados Unidos, otros. | <https://github.com/cov-lineages/pango-designation/issues/784> |
| 2022 | Recombinante | XAM | BA.1.1*BA.2.9 | Panamá, Estados Unidos, Perú, México, Indonesia, Chile, Colombia, otros. | <https://github.com/cov-lineages/pango-designation/issues/759> |
| 2022 | Ómicron | BG.6 | S: E1202Q  ORF1ab: V4266I | Perú, otros. | <https://github.com/cov-lineages/pango-designation/issues/836> |
| 2022 | Ómicron | BG.7 | ORF1a: F1632L ORF1a: F2321L 27630C>T | Perú. | <https://github.com/cov-lineages/pango-designation/issues/1157> |
| 2022 | Ómicron | DJ.1.2 | S: N460K | Perú, otros. | <https://github.com/cov-lineages/pango-designation/pull/1363> |
| 2022 | Ómicron | DJ.1.1.1 | S: K147N | Perú, Estados Unidos, Canadá, Chile, otros. | <https://github.com/cov-lineages/pango-designation/issues/1407> |
| 2022 | Ómicron | DJ.1.3 | S: N460K  ORF1a: T3287I 22924A>G | Perú, Estados Unidos, Colombia, otros. | <https://github.com/cov-lineages/pango-designation/issues/1409> |
| 2023 | Ómicron | BQ.1.1.50 | ORF1b: T792I  ORF1a: K669R 18159T>C | Perú, Chile, otros. | <https://github.com/cov-lineages/pango-designation/issues/1577> |
| 2023 | Ómicron | BQ.1.11.1 | ORF1a: E1633D | Perú, Estados Unidos, otros. | <https://github.com/cov-lineages/pango-designation/issues/1581> |
| 2023 | Ómicron | GN.1.4 | S: G181V | Perú, Estados Unidos, otros. | <https://github.com/cov-lineages/pango-designation/issues/2349> |

*: Solo se mencionan los principales países donde circulan, con un número de casos mayor a 10.
