# Supplementary material for: Implementation of a SARS-CoV-2 genomic surveillance network as a strategy to face other health challenges in Peru
Source: Rev Peru Med Exp Salud Publica. 2026 Mar 2;43(1):110–8. doi: 10.17843/rpmesp.2026.431.15048 (PMC13245991; doi:10.17843/rpmesp.2026.431.15048)
Supplement: Translation

# Translation: Implementación de una red de vigilancia genómica de SARS-CoV-2 como estrategia para enfrentar otros retos sanitarios en Perú

**Authors:** Carlos Padilla-Rojas, Verónica Hurtado, Wendy Lizarraga, Víctor Jimenez-Vasquez, Iris S. Molina, Luis Barcena-Flores, Alicia Nuñez, Nieves Sevilla, Steve V. Acedo-Lazo, Estela Huamán-Angeles, Princesa Medrano, Kelly Izarra, Alexander Fajardo, Vanesa Dominguez, Karla Vasquez, Orson Mestanza, Francisco Ascue, Henri Bailon, Marco Galarza-Pérez, Omar Caceres-Rey, Roger V. Araujo-Castillo, Natalia Vargas-Herrera, Joseph Huayra, Gloria Arotinco-Garayar, Priscila Lope-Pari, Johanna Balbuena-Torres, Nancy Rojas-Serrano

**Affiliations:** 1 Área de Innovación y Desarrollo, Centro Nacional de Salud Pública, Instituto Nacional de Salud, Lima, Perú.; 2 Unidad de Virología, Instituto Nacional de Salud, Lima, Perú.; 3 Unidad de Intervenciones Estratégicas, Instituto Nacional de Salud, Lima, Perú.; 4 Laboratorio de Referencia Nacional de Virus Inmunoprevenibles, Centro Nacional de Salud Pública, Instituto Nacional de Salud, Lima, Perú.

**Keywords:** Vigilancia en Salud Pública, SARS-CoV-2, Secuenciación de Nucleótidos de Alto Rendimiento, COVID-19

## Abstract

Presentamos una descripción completa de la implementación de la vigilancia genómica del SARS-CoV-2 durante la pandemia por COVID-19 en el Perú, y el impacto que tuvo al aplicar esta tecnología en otros patógenos importantes para la salud pública del país. Con el objetivo de resaltar los logros, desafíos y lecciones aprendidas que permitan fortalecer la respuesta ante los retos sanitarios presentes y futuros a nivel nacional, así como las decisiones en salud pública. Con la iniciativa de vigilancia genómica liderada por el Instituto Nacional de Salud se ha logrado secuenciar 48 691 genomas de SARS-CoV-2 procedentes de todas las regiones del país desde el año 2020 al 2024, logrando la identificación de ocho variantes del virus y la designación de 25 linajes nuevos dentro de las variantes Gamma, Delta y Ómicron. La vigilancia genómica de SARS-CoV-2 ha permitido identificar oportunamente variantes de interés epidemiológico, monitorizar la evolución del virus en tiempo real y utilizar esta información como herramienta para la toma de decisiones en salud pública. Esta experiencia posteriormente brindó las bases para vigilar otros patógenos que han causado emergencias sanitarias en el Perú y el mundo, como el dengue, influenza y viruela símica.

## INTRODUCCIÓN

La secuenciación del genoma completo (WGS, por sus siglas en inglés) de patógenos se ha consolidado en la actualidad como una herramienta fundamental en salud pública y constituye un pilar esencial en la epidemiología molecular. A diferencia de la epidemiología tradicional, basada en la descripción y seguimiento de los casos durante un brote o epidemia, la epidemiología molecular facilita la caracterización y detección temprana de variantes emergentes de patógenos, así como analizar sus patrones de transmisión, expansión y evolución en el tiempo ^1^. Sin embargo, la secuenciación genómica masiva es una actividad altamente especializada, por lo que, de manera previa a la pandemia, la experiencia en este tipo de iniciativas se centraba primordialmente en países desarrollados ^2^.

En Perú, de manera previa a la pandemia por COVID-19, el Instituto Nacional de Salud (INS) venía utilizando el método de Sanger para secuenciación de patógenos de relevancia en salud pública, como el virus de la rabia ^3^ y la varicela zoster ^4^. En el año 2010, se migró la plataforma hacia la secuenciación de siguiente generación (NGS, por sus siglas en inglés) para atender la necesidad suscitada inicialmente por la pandemia de influenza ^5^. En marzo del 2020, ante la necesidad de mejorar la detección de casos positivos de SARS-CoV-2, investigadores del Instituto Nacional de Salud (INS) lograron secuenciar el primer caso de SARS-CoV-2 en Perú mediante secuenciación metagenómica ^6^. Aunque se desarrolló un método *in house* basado en RT-qPCR, la capacidad de vigilancia genómica era limitada y dependía de proyectos financiados y presupuesto del INS. En dicha coyuntura, el INS conformó un equipo de vigilancia genómica para estudiar las mutaciones del virus para llevar a cabo la iniciativa de manera coordinada y efectiva.

Desde la confirmación del primer caso en Perú hasta fines del 2024, el INS impulsó la vigilancia genómica nacional, secuenciando exitosamente más de 48 mil genomas de SARS-CoV-2 hasta la fecha. El presente artículo destaca la experiencia, desafíos y logros en la vigilancia genómica de SARS-CoV-2 desde el 2020 hasta 2024 y cómo su implementación para responder a la pandemia de COVID-19 fue importante para otros desafíos a la salud pública que llegaron posteriormente. Con el objetivo de resaltar lecciones aprendidas que permitan fortalecer la respuesta ante los retos sanitarios presentes y futuros a nivel nacional, así como las decisiones en salud pública.

## DESCRIPCIÓN DE LA IMPLEMENTACIÓN DE LA RED DE VIGILANCIA GENÓMICA

El INS en coordinación con el Ministerio de Salud (MINSA) diseñó el plan de vigilancia genómica del SARS-COV-2 durante la emergencia sanitaria (Registro N°4814-2021) ^7^, el cual incluyó la coordinación con 25 laboratorios de referencia regional (LRR), laboratorios privados, Direcciones de Redes Integradas de Salud (DIRIS) y Direcciones o Gerencias Regionales de Salud (DIRESAS/GERESAS); abarcando desde la obtención y envío de muestras clínicas, hasta la descentralización de la prueba.

La implementación de la vigilancia genómica siguió las etapas: (i) elaboración y aprobación del protocolo, (ii) coordinación con los LRR, (iii) transporte de las muestras al INS, (iv) secuenciación genómica y análisis bioinformático, (v) publicación de genomas en base de datos públicas y (vi) reporte de resultados a las autoridades en salud para toma de decisiones en salud pública.

El proceso de vigilancia genómica requiere un tiempo de 15 días para la emisión de resultados, desde la detección de los casos hasta su caracterización. Este plazo se debe a la complejidad del procedimiento, las dificultades para el envío de muestras debido a la topografía del Perú y la edición manual de los genomas, una etapa crucial para asegurar la calidad de los datos. El flujo de trabajo completo se describe en la figura 1.

**Figura 1:** Flujo de trabajo de la vigilancia genómica del SARS-CoV-2 realizada en el INS del Perú. La colecta de muestras se realiza por los laboratorios de referencia de las 25 regiones del país, DIRIS, DIRESAS y los laboratorios de instituciones privadas.

INS: Instituto Nacional de Salud, MINSA: Ministerio de Salud, NETLAB: Sistema de Información de la Red Nacional de laboratorios de Salud Pública en el Perú, GISAID: Global Initiative on Sharing all Influenza Data.

Las muestras enviadas al INS se seleccionaban a nivel de los LRR, cuyos encargados elegían aquellas con diagnóstico positivo por RT-qPCR y un valor de Ct < 30. Si bien la mayoría de las muestras fue seleccionada de manera aleatoria, también se consideraba una priorización de acuerdo si la muestra procedía de un brote, de un viajero procedente del extranjero (con diagnóstico positivo en los últimos 14 días), o de una persona con reinfecciones, pacientes con síntomas inusuales y hospitalizados en la unidad de cuidados intensivos, llegando a 10 muestras por región. Se excluyeron las muestras con mala codificación, derrames, volumen insuficiente o incumplimiento de las normas de envío y conservación según la normativa vigente.

Para la extracción del material genético, se usaron plataformas automatizadas: Quick-DNA/RNA Viral MagBead Kit en el equipo automatizado OT-2 (Opentrons) para 48 muestras y Prepito Viral NA Body Fluid Kit en los equipos Chemagic® 360 y Prepito-D para 96 y 12 muestras, respectivamente. Después, se realizó la preparación de librerías con el kit COVIDSeq Test (Illumina Inc.), para luego proceder a la secuenciación en los equipos NextSeq 550, MiSeq y MiniSeq por paired-end con reads de 150 nt (300 ciclos). Los primers para la secuenciación se obtuvieron del Artic Network GitHub (https://github.com/artic-network/artic-ncov2019/tree/master/primer_schemes/nCoV-2019).

Los archivos obtenidos de la secuenciación fueron analizados para obtener el genoma completo del virus. Primero, se realizó un control de calidad utilizando el programa FastQC (https://www.bioinformatics.babraham.ac.uk/projects/fastqc/). Los reads fueron mapeados al genoma de referencia de SARS-CoV-2 Wuhan-Hu-1 (NC_045512) utilizando Samtools, BWA e iVar, mientras que la identificación de variantes y linajes se realizó con el Phylogenetic Assignment of Named Global Outbreak Lineages (Pangolin) (https://github.com/cov-lineages/pangolin) y las mutaciones nucleotídicas y aminoacídicas se detectaron con Nextclade (https://clades.nextstrain.org/). Los genomas fueron revisados manualmente con Aliview e IGV para posteriormente ser analizados a través de la construcción de filogenias con Nextstrain (https://nextstrain.org/). Los genomas obtenidos fueron depositados en la base de datos pública Global Initiative on Sharing All Influenza Data (GISAID) (https://gisaid.org/), para su uso por la comunidad científica. Asimismo, los resultados de la vigilancia genómica se registraron en el sistema Netlab.v2.0 (https://netlabv2.ins.gob.pe/) y se publicaron en la página web del INS (https://www.gob.pe/40668-conocer-la-secuenciacion-genomica-del-covid-19-en-el-peru), a través de una plataforma interactiva que proporciona datos sobre linajes y variantes circulantes, así como su distribución geográfica. Todos estos datos fueron compilados en Informes técnicos semanales emitidos al MINSA los cuales están disponibles en la plataforma digital única del estado peruano (https://repositorio.ins.gob.pe/).

Cabe señalar, en cuanto a las consideraciones administrativas de esta iniciativa, que las muestras fueron colectadas en el marco de la “Ficha de investigación Clinico-Epidemiologica de la COVID-19”, presente en la “Guía Técnica para Investigación y Control de Brotes de la COVID-19 en Instituciones con población cautiva” aprobada con Resolución Ministerial N° 440-2022/MINSA.

## ESCALAMIENTO DE LA VIGILANCIA GENÓMICA

Como se ha mencionado, durante el 2020 la capacidad para realizar secuenciación genómica en nuestro país era muy limitada y dependía principalmente del presupuesto interno del INS. En ese sentido, la posibilidad de realizar una vigilancia exhaustiva era muy limitada. En marzo de 2021, se logró desarrollar un plan nacional, que contemplaba el incremento de la capacidad para lograr secuenciar 384 muestras semanales de SARS-CoV-2 procedentes de todas las regiones del país. En 2022, esta capacidad aumentó a 912 muestras por semana debido a la contratación de más profesionales y adquisición de equipos. Esto se consideró necesario debido a que las regiones solicitaban información local, ya que para ese momento la dispersión genética del virus era amplia y cada región tenía dinámicas diferentes de transmisión.

Se debe resaltar que, en las regiones más alejadas, que requerían información genómica, pero cuya atención era complicada por las dificultades en el transporte de muestras, se implementó el laboratorio móvil Gen Maskaq, un laboratorio móvil adaptado en un vehículo motorizado, cuyo objetivo fue realizar la vigilancia genómica *in situ* en las regiones con un aumento importante de casos COVID-19*.* Este módulo prefabricado está equipado con una red eléctrica, ventilación, instalaciones sanitarias, mobiliario, además de contar con equipos de laboratorio necesarios para realizar el proceso de secuenciación genómica y áreas separadas, manteniendo el flujo unidireccional (Material Suplementario 1).

Además, a finales de 2021, el INS descentralizó la vigilancia genómica del SARS-CoV-2 a tres regiones clave: Piura, Cusco y Junín, en colaboración con sus LRR. Esto permitió capacitar un equipo de profesionales en la técnica de NGS, en cada una de estas regiones, lo que permitió procesar hasta 48 muestras semanales en cada LRR. La descentralización fue un éxito, gracias a los métodos de ensayo, protocolos y directivas sanitarias existentes, que facilitaron su replicación efectiva.

En total, el INS logró caracterizar 48 691 genomas provenientes de las 25 regiones del país hasta el 9 de abril del 2024 (figura 2), siendo Lima la región con el mayor número de casos secuenciados. Progresivamente, el INS incrementó su capacidad de secuenciación genómica, de producir 851 genomas en el 2020; al año siguiente, gracias al plan de vigilancia, se secuenciaron 12 246 genomas, cifra que aumentó exponencialmente a 29 157 genomas en 2022, esto debido en parte a la descentralización y escalamiento ya descrito de los procesos. En 2023, pese a la disminución de casos, se procesaron 5894 genomas y en 2024 se han secuenciado 543 genomas hasta abril de ese año (figura 3). Entre 2021 y 2022, los LRR secuenciaron un total de 4372 genomas.

**Figura 2:** Genomas de SARS-CoV-2 obtenidos producto de la vigilancia genómica realizada por el Instituto Nacional de Salud (INS) desde el año 2020 al 2024. Se detalla el número de genomas secuenciados por región (n) en el INS. Asimismo, se resalta con un icono de secuenciador en las regiones en donde se realiza la vigilancia genómica, detallando el número de genomas procesados por cada región (m).

**Figura 3:** Linajes de SARS-CoV-2 identificados por semana epidemiológica (SE) desde el año 2020 al 2024 en el Instituto Nacional de Salud del Perú. En la gráfica se ilustra la cantidad de linajes identificados desde la SE 10 del año 2020 a la SE 08 del 2024. Resalta los acontecimientos más importantes de la implementación de la vigilancia genómica durante la pandemia de COVID-19. Cada uno de los años está representado de un color diferente: 2020 (amarillo), 2021 (azul), 2022 (rojo), 2023 (verde) y 2024 (anaranjado).

## PERTENENCIA A REDES INTERNACIONALES

Los informes técnicos de vigilancia genómica de SARS-CoV-2 están disponibles en: https://www.gob.pe/institucion/ins/colecciones/15034-informes-tecnicos-de-variantes-y-linajes-del-virus-sars-cov-2. En su momento constituyeron insumos importantes para la toma de decisiones del MINSA. Así como se fue formando una red de análisis genómico entre el INS y algunas regiones al interior del país, el Perú participó en las redes internacionales de análisis genómico del continente, en particular Red Regional de Vigilancia Genómica de COVID-19 en la Región de las Américas (COVIGEN), y la Red Regional de Vigilancia Genómica de Virus Respiratorios (RESVIGEN), entre otras.

En este sentido, la divulgación científica ha sido fundamental para crear redes de colaboración y promover la investigación (Material Suplementario 2), en eventos organizados por el INS de Colombia, Red Federal de Genómica y Bioinformática en Argentina, Organismo Andino de Salud y el Convenio Hipolito Unanue (ORAS-CONHU), Seoul Clinical Laboratories de Corea del Sur y la Organización Panamericana de la Salud.

## IMPACTO DE LA SECUENCIACIÓN MASIVA DEL SARS-CoV-2 EN EL PERÚ

Hasta la fecha, en el Perú se han generado 55 280 genomas de SARS-CoV-2, de los cuales el 88% es producto del trabajo del INS, lo que lo convierte, según GISAID, en el segundo país en Sudamérica con mayor secuenciación de genomas reportados de SARS-CoV-2, solo superado por Brasil ^8^.

A lo largo del proceso de implementación de vigilancia genómica del SARS-CoV-2 en Perú, el INS detectó la circulación en el país de 8 variantes del SARS-CoV-2 desde el 2020 hasta el 2024: 4 variantes de preocupación (VOC) Alfa (B.1.1.7), Gamma (P.1), Delta (B.1.617) y Ómicron (B.1.1.529), 2 variantes de interés (VOI) Lambda (C.37) y Mu (B.1.621) y 2 variantes bajo vigilancia (VUM) Épsilon (B.1.429) y Zeta (P.2.). Cabe resaltar que a finales de 2022 se detectó por primera vez casos de linajes recombinantes dentro de la variante Ómicron. Algunos linajes como DJ.1, GN.1, JD.1, BA.2.86, JN.1.42 han causado preocupación en Perú debido a su alta prevalencia y rápida dispersión, sin embargo, no han sido designados como variantes por la Organización Mundial de la Salud (OMS). Un mayor detalle sobre las variantes y linajes de SARS-CoV-2 detectados se encuentra en la figura 4.

**Figura 4:** Representación gráfica de las principales variantes y linajes de SARS-CoV-2 identificadas en Perú desde el año 2020 al 2024. Se ilustra la evolución del virus SARS-CoV-2 y la aparición de las variantes de preocupación, interés y bajo vigilancia en Perú. La descendencia directa de un linaje se representa en líneas completas, mientras que las líneas punteadas denotan que existen múltiples linajes intermedios en su evolución. Los linajes o variantes con mayor prevalencia o causantes de olas epidemiológicas son representados con borde azul.

Al inicio de la pandemia, se detectó una amplia circulación del linaje B.1.1, el cual fue el más persistente, especialmente en Lima y Callao ^9^. Este linaje daría origen a la primera variante de preocupación designada por la OMS, denominada Alfa (B.1.1.7), la cual fue más predominante en Europa, sólo unos pocos casos fueron reportados en Perú ^(^^10^, relacionado con la limitada caracterización genómica y con la llegada de otras variantes de interés como Lambda (C.37) y de preocupación como Gamma (P.1) que se dispersaron durante la segunda ola epidemiológica en Perú ^11^^,^^12^. Durante este periodo del 2021 se logró detectar por primera vez varios sublinajes de la variante Gamma, denominados P.1.12, P.1.12.1 y P.1.7.1, circulantes en países como Estados Unidos, Brasil y Chile.

En el segundo trimestre del 2021 emergieron la VOC Delta (B.1.617.2) y la VOI Mu (B.1.621). Durante este periodo se detectó seis sublinajes en la amplia diversidad de Delta, estos fueron designados como AY.26.1, AY.3.2, AY.102.2, AY.102.1, AY.119.1 y AY.25.1.1, aparentemente habiéndose generado algunos de ellos en Perú, mientras que otros se dispersaron a Chile, Estados Unidos, Brasil, Colombia, España y Costa Rica. A finales del 2021 Delta fue desplazado por la VOC Ómicron (B.1.1.529) causante de la tercera ola epidemiológica en Perú a inicios del 2022.

En 2022, se observó una amplia diversidad y desplazamiento secuencial de Ómicron, destacando sus linajes BA.1, BA.2, BA.4 y BA.5, los cuales fueron detectados en Perú de manera sucesiva. Este desplazamiento entre linajes, observado a nivel mundial, se debe a la competencia entre variantes con ventajas en transmisibilidad y/o un mayor escape inmunológico ^13^. A partir de BA.5 vimos la emergencia de sus descendientes como DJ.1, DJ.2 y BQ.1 ^14^. A nivel global, BA.5 ha demostrado una evolución convergente significativa, con numerosas mutaciones compartidas en el gen spike ^15^, además de ser altamente transmisible y capaz de evadir la inmunidad previa ^16^. Dentro de Ómicron detectamos 15 linajes descendientes de BA.1, BA.2, DJ.1, BQ.1, GN.1. A finales de 2022 se detectó la presencia del linaje recombinante XBB de ómicron, producto de la combinación de los linajes BA.2.10.1 y BA.2.75. Aunque el recombinante XBB muestra una mayor capacidad de evasión inmune en comparación con los linajes anteriores de Ómicron, no hay evidencia de que cause una enfermedad más grave (https://boletin.ins.gob.pe/informacion-institucional-n6/). En 2023, el recombinante XBB predominó hasta la semana epidemiológica SE 06, cuando comenzó a descender lentamente, siendo desplazado por los linajes XBB.1.5 y sus descendientes. A finales de 2023, el linaje JN.1, descendiente directo de BA.2.86.1 comenzó a incrementarse rápidamente y manteniéndose hasta la SE 08 del 2024, desde donde sólo prevalecieron los sublinajes JN.1 y JN.1.42 como los más frecuentes.

En total, el equipo de Vigilancia Genómica del INS propuso 62 linajes nuevos de SARS-CoV-2 desde el año 2021 hasta 2024, logrando que 25 sean aprobados y designados por el comité internacional Pangolin (https://github.com/cov-lineages/pango-designation). Estos pertenecen a Gamma (P.1.12, P.1.12.1, P.1.7.1), Delta (AY.26.1, AY.3.2, AY.102.2, AY.102.1, AY.119.1, AY.25.1.1), Ómicron (BA.1.22, BC.2, BA.1.15.3, BA.2.53, BG.1, BA.2.60, BG.3, BG.6, BG.7, DJ.1.2, DJ.1.1.1, DJ.1.3, BQ.1.1.50, BQ.1.11.1, GN.1.4) y Recombinante (XAM). Sus características se describen detalladamente en el Material Suplementario 3. Esto refleja la contribución del INS al conocimiento de la genómica global del virus.

## VIGILANCIA GENÓMICA DE OTROS PATÓGENOS DE INTERÉS EN SALUD PÚBLICA

Luego de la pandemia por SARS-CoV-2, el Perú ha enfrentado brotes de diversas enfermedades infecciosas, incluyendo dengue, influenza y viruela símica. Tras haber implementado capacidades nacionales para realizar la vigilancia genómica de patógenos, se pudo aplicar las habilidades adquiridas a otras epidemias.

### Viruela símica

En 2022, la OMS declaró un brote del virus de viruela símica, el cual infectó a 3800 personas en Perú y causó 20 muertes en 21 regiones del país, mayoritariamente en personas con un sistema inmunitario debilitado ^17^. Como respuesta al brote, el INS a través del equipo de vigilancia genómica caracterizó el genoma del primer caso confirmado de la viruela símica en Perú, en junio del 2022, posteriormente se continuó con la secuenciación de los casos confirmados a nivel nacional, la procedencia de los genomas caracterizados en mayor porcentaje fue de las regiones de Lima, Callao, La Libertad y Arequipa. Durante el brote y los años posteriores de vigilancia, el INS fue la única institución peruana que realizó la caracterización genómica de viruela símica, reportando 575 genomas, los cuales pertenecen al clado IIb, siendo el linaje predominante B.1.6, nativo del Perú, que se dispersó ampliamente en todo el país (https://github.com/mpxv-lineages/lineage-designation/issues/9). El hecho de haber producido un linaje propio del país, y que este haya sido el predominante, indica que posiblemente la epidemia en Perú tuvo una magnitud mayor de la reportada.

### Influenza

En 2023, se inició la secuenciación sistemática del virus de la influenza, como parte de la implementación de un sistema de monitoreo genómico, dada la alta capacidad del virus para experimentar mutaciones y reordenamientos genéticos. Los primeros resultados permitieron identificar la circulación de virus de influenza tipo A, específicamente los subtipos A(H1N1)pdm09 y A(H3N2), así como linaje B/Victoria perteneciente a influenza tipo B. Por otro lado, los análisis filogenéticos revelaron patrones evolutivos del virus a lo largo del tiempo, evidenciando la formación de clados genéticos específicos según el periodo de circulación. Actualmente, se realiza una vigilancia genómica trimestral del virus influenza humana.

En el contexto de la emergencia sanitaria por influenza aviar de alta patogenicidad (H5N1) registrada en el Perú durante 2023, se logró la caracterización genética de 13 aislamientos del subtipo H5N1, obtenidos a partir de aves y mamíferos silvestres, principalmente en ecosistemas costeros. Los análisis filogenéticos permitieron establecer que todas las secuencias virales pertenecieron al clado 2.3.4.4b. Este trabajo se desarrolló en coordinación con el Servicio Nacional Forestal y de Fauna Silvestre y el Servicio Nacional de Sanidad Agraria, entidades responsables de la recolección y el envío de las muestras biológicas para su procesamiento molecular ^18^.

### Dengue

El INS ha estado participando en la respuesta a los brotes del virus dengue del 2023, logrando caracterizar 289 muestras. Sin embargo, fue en el año 2024, que el INS implementó la vigilancia genómica del virus del dengue con una frecuencia trimestral. En el marco de la vigilancia, 1184 muestras fueron caracterizadas mediante NGS en el año 2024. Esta implementación ha permitido realizar un monitoreo en tiempo real de los genotipos y mutaciones presentes en este virus.

Los primeros resultados de la vigilancia han logrado identificar a los genotipos circulantes dentro de los 3 serotipos de dengue: el genotipo V (DENV-1), genotipo Cosmopolitan (DENV-2) y genotipo III (DENV-3). Estos resultados coinciden con lo reportado previamente desde el 2018 a 2022 ^19^. Durante el 2023, se identificó el genotipo Cosmopolitan del serotipo DENV-2 que presentaba una distribución geográfica amplia en diferentes regiones del país en mayor proporción de casos. Sin embargo, se evidenció un cambio por el incremento del genotipo III del serotipo DENV-3 y particularmente el crecimiento de un nuevo clado, hallazgo reportado en el segundo trimestre del 2024.

A diferencia de la identificación de serotipos que se realiza mediante pruebas serológicas, hasta el momento la caracterización de los genotipos y linajes de dengue solo se puede realizar por secuenciación genómica y un posterior análisis filogenético que evalúe la formación y distribución de los clados. Continuar con la vigilancia genómica del virus del dengue a nivel nacional nos permitirá una mejor comprensión de su comportamiento epidemiológico en el país.

## RECOMENDACIONES

A partir de la experiencia adquirida con la vigilancia genómica de SARS-CoV-2 se debe mantener el funcionamiento adecuado de los Laboratorios de Referencia Regional y del INS que realizan vigilancia genómica a través de su adecuado financiamiento y personal entrenado, para poder enfrentar las futuras emergencias sanitarias. Consideramos que es vital mantener el uso de la secuenciación de siguiente generación para identificar variantes y linajes de cepas virales que sean un riesgo para la salud pública nacional.

En ese sentido, debemos impulsar la descentralización de la secuenciación a más regiones del país con el objetivo de que puedan realizar una vigilancia oportuna de los patógenos circulantes en su región y atiendan sus problemas de salud pública regional. Es importante destacar que toda la información genómica recopilada producto de esta vigilancia debe estar disponible y de acceso libre a la comunidad científica para beneficio de toda la población.

## CONCLUSIONES

A partir de la implementación de la vigilancia genómica de SARS-CoV-2, en el 2020 se ha logrado monitorear el ingreso de nuevas variantes y el surgimiento de linajes virales, logrando identificar la circulación de 465 linajes en nuestro territorio y registrando 25 propios de nuestro país. Esta vigilancia genómica ha demostrado su utilidad durante la pandemia de COVID-19, permitiendo comprender la dispersión y evolución del virus en nuestro país.

La implementación de la vigilancia para SARS-CoV-2 fue la respuesta a la necesidad de tener información más precisa sobre los patógenos, por lo cual se consideró necesario ampliarla a otras enfermedades causantes de brotes. Es así como inicialmente se implementó para viruela símica, obteniendo como resultado la asignación de 575 muestras dentro del clado IIb e identificando que el linaje B.1.6, nativo del Perú, fue el más predominante en el país. Posteriormente, la vigilancia se utilizó durante la identificación del riesgo de la introducción de la Influenza aviar, encontrándose que el clado 2.3.4.4b del grupo altamente patogénico se encontraba circulando en las aves y mamíferos de la costa peruana. Por último, en la epidemia de dengue, se identificó a los genotipos V (DENV-1), Cosmopolitan (DENV-2) y III (DENV-3), y se analizó la sucesión de serotipos; observando que el serotipo DENV-2 con mayor frecuencia en el año 2023 fue desplazado por el serotipo DENV-3 en el 2024.

El principal desafío en emergencias sanitarias por epidemias, al momento de la implementación de la vigilancia genómica, es asegurar los fondos necesarios para implementar un sistema de envío de muestras, insumos para secuenciación, personal capacitado y mejoramiento de infraestructura. La experiencia adquirida en la vigilancia genómica del SARS-CoV-2 en el Perú, pudo servir como modelo para subsiguientes implementaciones durante brotes o epidemias como las mencionadas. Es crucial estandarizar y unificar la información de salud en el país y conectarla con el sistema de vigilancia genómica para mejorar la capacidad de respuesta. La amenaza de pandemias ha fomentado una colaboración fluida entre sectores, lo cual debe ser promovido y priorizado para enfrentar nuevas amenazas a la salud pública.

Consideramos que la implementación de la vigilancia genómica de patógenos de interés en salud pública ha sido un éxito en nuestro país. Producto de todos los resultados obtenidos en la implementación es que se han recibido múltiples reconocimientos. En 2023, la práctica de vigilancia genómica fue reconocida como una Buena Práctica en Gestión Pública por la organización Ciudadanos al Día y obtuvo el tercer lugar en el XVIII Encuentro Nacional de Experiencias en Mejoramiento de la Calidad en Salud organizado por el MINSA.

A pesar del éxito de la vigilancia genómica, persisten múltiples desafíos que deben ser abordados para mejorar la capacidad de respuesta ante una próxima emergencia sanitaria. Desde el inicio de la pandemia, se ha evidenciado que la topografía del país complica el envío de muestras desde diversos distritos y provincias, lo que ha provocado la demora del envío de muestras al INS. Este retraso se ha visto agravado por temas logísticos y presupuestales, lo que hace aún más urgente la necesidad de contar con un sistema más eficiente para el envío de muestras. Asimismo, es necesario sensibilizar a la población sobre la importancia de la vigilancia genómica, ya que esta permite identificar nuevas variantes y monitorear su evolución, lo que ayuda a implementar estrategias de control efectivas y fortalecer la respuesta ante futuras crisis sanitarias. Es por ello sumamente importante asegurar el presupuesto necesario para continuar con esta actividad que es crucial, para poder seguir implementándola en otras amenazas a la salud pública por venir.
